# Physiological changes associated with copper sulfate-induced nausea and retching in felines

**DOI:** 10.3389/fphys.2023.1077207

**Published:** 2023-01-19

**Authors:** Charles P. Murphey, Jonathan A. Shulgach, Pooja R. Amin, Nerone K. Douglas, John P. Bielanin, Jacob T. Sampson, Charles C. Horn, Bill J. Yates

**Affiliations:** ^1^ Department of Otolaryngology, University of Pittsburgh School of Medicine, Pittsburgh, PA, United States; ^2^ Department of Neuroscience, University of Pittsburgh, Pittsburgh, PA, United States; ^3^ Mechanical Engineering Department, Carnegie Mellon University, Pittsburgh, PA, United States; ^4^ UPMC Hillman Cancer Center, Pittsburgh, PA, United States; ^5^ Department of Medicine, University of Pittsburgh School of Medicine, Pittsburgh, PA, United States; ^6^ Department of Anesthesiology and Perioperative Medicine, University of Pittsburgh School of Medicine, Pittsburgh, PA, United States

**Keywords:** nausea, vomiting, gastric electromyogram, copper sulfate, respiratory activity detection, heart rate

## Abstract

Nausea is a common disease symptom, yet there is no consensus regarding its physiological markers. In contrast, the process of vomiting is well documented as sequential muscular contractions of the diaphragm and abdominal muscles and esophageal shortening. Nausea, like other self-reported perceptions, is difficult to distinguish in preclinical models, but based on human experience emesis is usually preceded by nausea. Here we focused on measuring gastrointestinal and cardiorespiratory changes prior to emesis to provide additional insights into markers for nausea. Felines were instrumented to chronically record heart rate, respiration, and electromyographic (EMG) activity from the stomach and duodenum before and after intragastric delivery of saline or copper sulfate (CuSO_4_, from 83 to 322 mg). CuSO_4_ is a prototypical emetic test agent that triggers vomiting primarily by action on GI vagal afferent fibers when administered intragastrically. CuSO_4_ infusion elicited a significant increase in heart rate, decrease in respiratory rate, and a disruption of gastric and intestinal EMG activity several minutes prior to emesis. The change in EMG activity was most consistent in the duodenum. Administration of the same volume of saline did not induce these effects. Increasing the dose of CuSO_4_ did not alter the physiologic changes induced by the treatment. It is postulated that the intestinal EMG activity was related to the retrograde movement of chyme from the intestine to the stomach demonstrated to occur prior to emesis by other investigators. These findings suggest that monitoring of intestinal EMG activity, perhaps in combination with heart rate, may provide the best indicator of the onset of nausea following treatments and in disease conditions, including GI disease, associated with emesis.

## 1 Introduction

Nausea is an important defense mechanism against poisoning, is a symptom of a variety of conditions, such as gastrointestinal (GI) disease and vestibular disorders, and it is a common side effect of many clinical treatments, such as cancer chemotherapy, inhalational anesthesia, and opioid pain medications (Golding and Gresty, 2015; Piechotta et al., 2021; Weibel et al., 2021). Nausea is also difficult to treat, especially in chronic conditions where patients are often provided anti-emetics, which are less effective for the control of nausea (Koch et al., 2016; Lacy et al., 2018). In contrast to other conditions, such as pain and anxiety, the physiological indicators of nausea are not well established. Based on human experience, emesis, which is produced through a well-characterized sequence of muscle contractions (Grelot and Miller, 1994), is usually preceded by nausea.

Changes in cardiac and GI myoelectric responses are commonly reported correlates of nausea in humans, specifically related to emetogenic platinum-based chemotherapy (using drugs such as cisplatin), GI diseases, and motion sickness (Morrow et al., 2000; Himi et al., 2004; Carson et al., 2022). Increased heart rate, decreased heart rate variability, and changes in gastric myoelectric activity have been reported to be associated with nausea (Morrow et al., 2000; Himi et al., 2004; Kim et al., 2011; LaCount et al., 2011). Several preclinical studies using the most common emetic test species (dog, cat, and ferret) have tracked the temporal associations between emesis and these responses. In dogs, cisplatin chemotherapy and intragastric copper sulphate (CuSO_4_) disrupt intestinal and gastric myoelectric activity (Lee et al., 1985; Chey et al., 1988; Ando et al., 2014). In cats, centrally acting emetic agents, particularly morphine and apomorphine, disrupt intestinal myoelectric activity associated with emesis (Stewart et al., 1977). In addition, studies using ferrets showed simultaneous changes in cardiac and GI myoelectric measures following emetic treatments; for example, cisplatin treatment produced gastric myoelectric disruption, an increase in heart rate, and a decrease in heart rate variability (Lu et al., 2017). Limitations of these prior studies include a lack of simultaneous measures in each experiment (e.g., intestinal, gastric, and cardiorespiratory recordings). In addition, most failed to use an acute emetic stimulus, such as intragastric CuSO_4_, to permit temporal associations between physiological measures and the onset of emesis.

The focus of the current study was to examine changes in cardiorespiratory, gastric and intestinal myoelectric signals preceding emesis, and, therefore, to ascertain potential markers for nausea. We used the cat model in the current work because of its well-documented physiologic responses during emesis (Makale and King, 1992; Hickman et al., 2008), ease of surgical electrode placement, and adaptability to behavioral training. Importantly, we trained animals to remain sedentary while loosely restrained to limit the occurrence of electrical artifacts. We also used intragastric CuSO_4_ as the emetic test stimulus because copper and its derivatives are known to elicit nausea in humans (Pizarro et al., 1999; Araya et al., 2001). CuSO_4_ rapidly triggers emesis in cats and other preclinical models primarily by activating the GI vagus nerve-to-brain pathway (Wang and Borison, 1951; Brizzee and Marshall, 1960; Makale and King, 1992; Horn et al., 2014). The acute action of this agent allowed us to monitor a variety of physiologic parameters prior to emesis during a controlled recording session. Before and following CuSO_4_ treatment we recorded heart rate, respiration rate, and gastric and duodenal myoelectric responses using methodology developed through our work in ferrets (Nanivadekar et al., 2019; Shulgach et al., 2021). The dose of CuSO_4_ was additionally varied between animals so we could determine whether increasing the dose altered physiologic responses prior to emesis.

A preprint of this article was posted on the BioRxiv preprint server (Murphey et al., 2022).

## 2 Methods and materials

### 2.1 Animals and husbandry

All experimental procedures on animals were approved by the University of Pittsburgh’s Institutional Animal Care and Use Committee, in accordance with the National Research Council’s Guide for the Care and Use of Laboratory Animals (National Research Council, 2011). Experiments were performed on 10 antibody profile defined and specific pathogen free domestic shorthair cats (6 male, 4 female) obtained from Marshall BioResources (North Rose, New York, United States of America). Animals were provided commercial cat food and water *ad libitum* and were housed under 12 h light/dark cycles. Characteristics about the animals and other experimental parameters are provided in Table 1.

**TABLE 1:** Characteristics of animals used in the experiments, as well as experimental parameters.

Animal id	Sex	# Acclimation days prior to surgery	# Days following surgery and prior to final experiment	Weight at final experiment (kg)	Total number of recording sessions (control + final experiment)
C1-21	F	65	48	3.78	29
C2-21	F	92	30	3.44	11
C3-21	F	51	57	2.75	32
C11-21	F	36	43	3.70	23
C13-20	M	154	50	4.70	23
C14-20	M	133	32	4.10	19
C15-20	M	138	46	5.40	26
C20-20	M	141	32	3.91	19
C48-19	M	112	45	5.00	27
C49-19	M	114	29	4.40	17

### 2.2 Acclimation and surgical procedures

**FIGURE 1:**
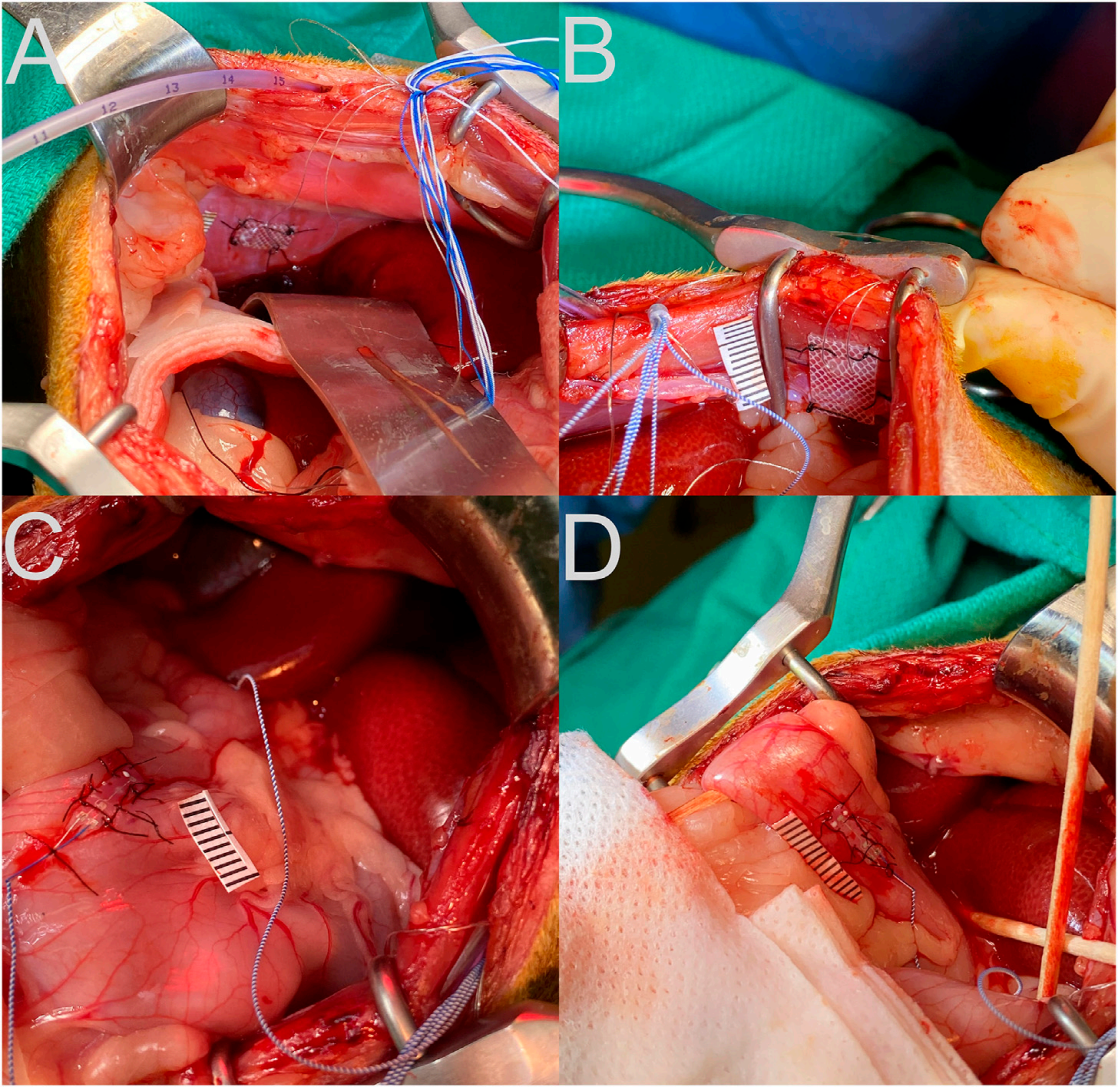
Placement of recording electrodes on the diaphragm **(A)**, abdominal musculature **(B)**, stomach antrum **(C)**, and upper duodenum **(D)**. A ruler with 1 mm hatch marks is placed next to the electrode in each panel.

As in our prior experiments (Patel et al., 2018; Bielanin et al., 2020; Miller et al., 2020), animals were gradually acclimated over at least 5 weeks for 1-h of confinement in a nylon restraint bag attached to a recording platform using Velcro straps. Subsequently, an aseptic recovery surgery was performed in a dedicated operating suite as described below to attach two-contact platinum-iridium 90/10 electrodes in a silicone patch (Micro-leads Inc, Somerville, MA, United States), similar to those used in prior studies (Nanivadekar et al., 2019; Shulgach et al., 2021), to the proximal duodenum and gastric antrum, on the ventral surface (Figure 1). In addition, custom-fabricated electrodes consisting of flexible insulated wire (Cooner Wire, Chatsworth, CA United States) embedded in medical grade silicon (Bentec Medical, Woodland, CA United States) were attached to the diaphragm and abdominal muscles (Figure 1).

For the surgery, animals were initially anesthetized with an intramuscular injection of ketamine (15 mg/kg) and acepromazine (0.5 mg/kg) to permit the insertion of an endotracheal tube. Subsequently, anesthesia was maintained with 1%–2% isoflurane vaporized in oxygen delivered through that tube. Heart rate, respiration rate, and blood oxygen saturation, as well as withdrawal reflexes, were monitored throughout the surgery to guide the concentration of isoflurane that was delivered. A heating lamp and pad were used to keep the animal’s body temperature near 38°C. Saline was administered intravenously throughout the surgery to maintain hydration.

A laparotomy was performed to expose the abdominal organs. Electrodes were secured to the gastric antrum, duodenum, costal diaphragm, and inner surface of the transversus abdominis using sutures through the silicon patch surrounding the recording leads, as shown in Figure 1. A small incision was made through the lateral edge of the gastric fundus for introduction of an 8-french intragastric pediatric feeding tube. The tube was secured in place using a purse-string silk suture and Vetbond adhesive (3M, St. Paul, MN United States). The abdominal cavity was lavaged with sterile saline prior to separately closing the abdominal muscles and skin using sutures. Connectors and leads from the electrodes and the distal end of the intragastric catheter were routed subcutaneously to the neck using a trocar. A small area of the skull was exposed, self-tapping screws were inserted, and the connectors were attached to the skull using Palacos bone cement (Heraeus Medical, Hanau, Germany).

A fentanyl transdermal patch (25 μg/h delivery) was attached to the shaved skin on one leg, which provided analgesia for 72 h. In addition, 0.3 mg/kg Meloxicam was provided subcutaneously at the end of surgery and re-administered daily upon veterinary recommendation to alleviate indicators of pain or discomfort. The long-acting antibiotic Convenia (Zoetis, New York, NY United States) was administered during surgery (8 mg/kg subcutaneously).

Following a ∼2-week post-surgical recovery period, an additional period of acclimation for restraint occurred (see Table 1) prior to commencement of data recording.

### 2.3 Data recording

**FIGURE 2:**
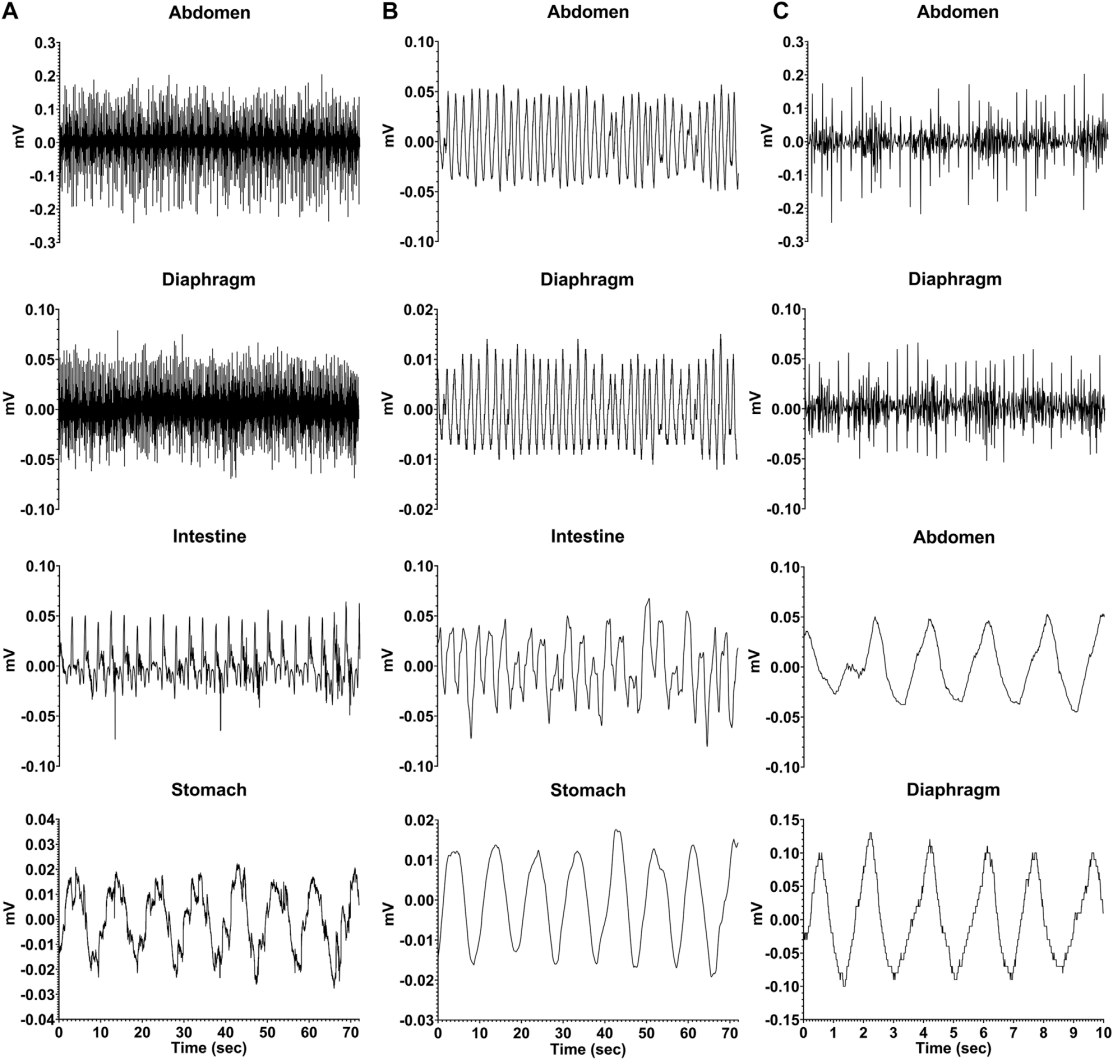
Recordings from one animal (C2-21) during a baseline trial where neither saline nor CuSO_4_ was administered. **(A)** raw EMG recordings from the abdominal musculature, diaphragm, intestine (duodenum), and stomach. **(B)** same recordings, smoothed with a 0.2 s time constant. **(C)** diaphragm and abdominal muscle activity for a shorter time period (first 10 s of traces in A) so that details of recordings are evident. The top two traces are raw EMG recordings, while the bottom two traces depict the same data smoothed with a 0.2 s time constant.

During recording sessions, signals from the connectors for the electrodes attached to the diaphragm and abdominal muscles were led into Model 1800 AC differential amplifiers (A-M Systems, Sequim, WA United States), amplified 100 times and filtered with a bandpass of 10–10,000 Hz. Signals from the electrodes positioned on the stomach and intestine were led into Model 3000 AC/DC differential amplifiers (A-M Systems) with the high-pass filter in DC mode, and amplified 50 times. The signals were digitized and recorded at 1 kHz by a Micro1401 mk2 data collection system and Spike-2 version 7 software (Cambridge Electronic Design, Cambridge, United Kingdom). Examples of recordings are provided in Figure 2.

A number of recording sessions were performed to validate that recordings were stable, and in at least one of those sessions 20 mL of saline was infused into the stomach following an 12–18 h fast, while recordings from implanted electrodes were made to determine heart rate, respiration rate, and the frequency of stomach and duodenal slow wave activity. Following the collection of these baseline data, a terminal recording session occurred when a single dose of 83–332 mg of CuSO_4_ dissolved in 20 mL saline (see Table 2 for the dose in each animal) was infused into the stomach, through oral gavage in three cases and the intragastric tube in the other seven animals. Prior studies showed that CuSO_4_ doses of 83 mg reliably produce physiologic changes in felines (Sugiyama et al., 2011; Suzuki et al., 2012); larger doses were also used to determine if they elicited similar responses. Animals were fasted overnight prior to this experimental session. Oral gavage occurred in cases where the intragastric catheter became blocked prior to the final experimental session. As during the control session when saline was infused into the stomach, activity recorded from electrodes was used to determine heart rate, respiration rate, and the frequency of stomach and duodenal slow wave activity prior to and following CuSO_4_ administration. Stereotyped contractions of the diaphragm and abdominal musculature were used to determine the timing of emetic episodes (Yates et al., 2014). These data were employed to ascertain whether prodromal changes in physiological parameters predict an emetic episode evoked by CuSO_4_.

**TABLE 2:** Responses of animals to CuSO4 administration.

Animal id	Sex	Route of CuSO4 administration	CuSO4 Dosage (mg)	Time to first retch (min)	Duration of retching episode (sec)
C1-21	F	Intragastric	166	25	11.2
C2-21	F	Oral	332	4	7.2
C3-21	F	Oral	332	33	5.3
C11-21	F	Oral	332	6	8.9
C13-20	M	Intragastric	83	14	11.8
C14-20	M	Intragastric	332	3	7.5
C15-20	M	Intragastric	332	33	13.5
C20-20	M	Intragastric	83	6	10.1
C48-19	M	Intragastric	166	2	7.0
C49-19	M	Intragastric	166	10	6.2

During control recording sessions, saline was administered 10 min following the onset of the recording, so baseline physiologic parameters could be determined. Recordings continued for 60 min. A similar recording strategy was used during the final session when CuSO_4_ was administered. Following the conclusion of recordings in this session, animals were anesthetized by an intramuscular injection of ketamine (20 mg/kg) and acepromazine (0.2 mg/kg) followed by an intraperitoneal injection of pentobarbital sodium (40 mg/kg). Animals were then transcardially perfused with saline followed by 4% paraformaldehyde fixative. The placement of recording electrodes was evaluated by dissecting the abdominal cavity and extracting the diaphragm and GI tract for imaging.

### 2.4 Data analysis

Offline analysis of recordings was performed using Spike-2 version 9, MATLAB (Mathworks, Natick, MA United States), and Python (Python Software Foundation) software. As an initial step, DC drift was removed from all traces. Signals from respiratory muscles were smoothed (time constant of 0.2 s) and/or rectified for some analyses. Myoelectric signals recorded from the stomach and intestine electrodes were processed using a bandpass second order Butterworth filter with a 0.05–0.5 Hz (3–30 cycles per minute, cpm) cutoff range, and then downsampled to 10 Hz. Because the head was not restrained, some non-physiological high amplitude artifacts lasting 1–20 s were observed during head movements. These artifacts were cleaned by implementing a blanking window around the artifact period with 1 s before and after the event and replacing the blanked portion with a linearly interpolated line that spanned the window. The power spectrum for myoelectric signals was calculated by computing the fast Fourier transform (FFT, 0.014 cpm bin size). The dominant frequency was determined by finding the median frequency of a power spectral density estimate at each 1-min segment throughout the length of the signal. Statistical analyses were conducted and some figures were plotted using Prism 9 software (GraphPad software, San Diego, CA United States). Data are presented as means ± one standard deviation.

## 3 Results

Figure 2 shows raw and processed data recorded during a baseline run in one animal. Column A illustrates raw traces, and in Column B the same traces were smoothed (time constant of 0.2 s). Column C expands the first 10 s of respiratory muscle activity shown in column A so that details of traces are evident; the top two traces show raw activity, and the bottom two are smoothed activity (time constant of 0.2 s). Both the electrocardiogram and respiratory muscle electromyographic (EMG) activity were evident in recordings from the diaphragm and abdominal muscles, such that these traces could be used to determine both heart rate and respiration rate. In addition, slow wave activity was evident for both the stomach (frequency of ∼0.1 Hz or 6 cpm) and duodenum (frequency of ∼0.3 Hz or 18 cpm), which occurred at a different rate than respiration (frequency of ∼0.6 Hz or 36 cpm) indicating it was not an artifact related to abdominal movements during breathing. Heart rate, respiration rate, and frequency of stomach and duodenal slow wave activity are shown in Table 3, determined for each animal during a 60-min period of a baseline recording. Some physiologic parameters could not be determined in a few animals due to low-quality recordings from particular electrodes. The average baseline heart rate for all animals was 163 ± 26 beats per minute, and the average respiratory rate was 34 ± 16 breaths per minute. Slow wave activity occurred at 6 ± 1 cpm in the stomach and 20 ± 1 cpm in the duodenum.

**TABLE 3:** Baseline physiological measurements for each animal. X—data not acquired.

Animal id	Average heart rate (BPM)	Average respiration rate (BPM)	Average intestine slow wave activity (cpm)	Average stomach slow wave activity (cpm)
C1-21	160	x	x	x
C2-21	163	32	19	5
C3-21	232	73	20	8
C11-21	150	23	18	7
C13-20	139	41	19	4
C14-20	161	21	18	6
C15-20	145	22	19	6
C20-20	142	20	23	6
C48-19	182	47	21	6
C49-19	153	30	20	5

**FIGURE 3:**
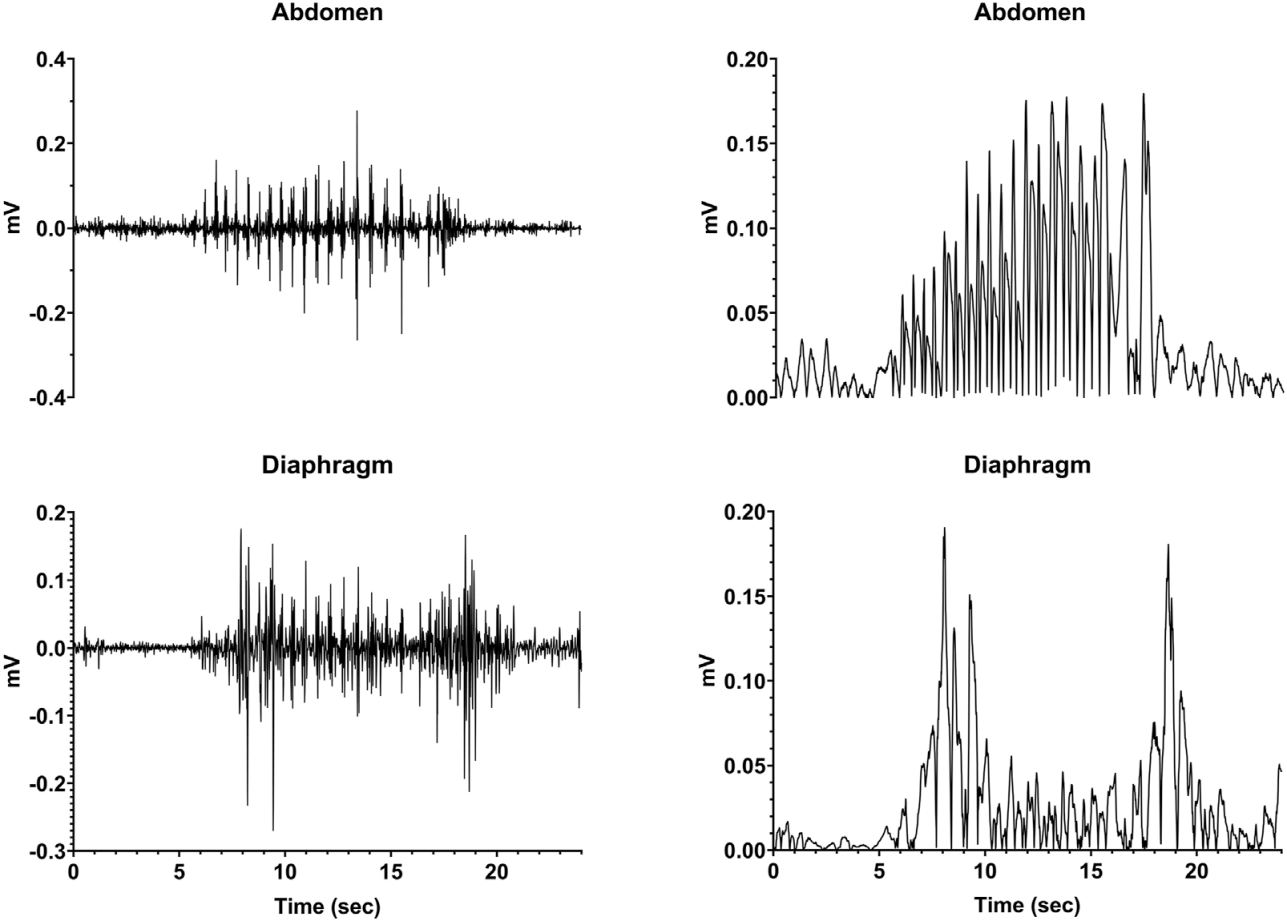
Recordings of diaphragm and abdominal muscle activity during a retching episode (continuous series of retches). The left traces are raw EMG activity, whereas the recordings are rectified and smoothed (time constant of 0.2 s) in the right panels.

Following the administration of CuSO_4_, we visually observed the animals for indications of retching and compared these observations to recordings from the electrodes. In all cases, a retching period occurred as a series of contractions that was accompanied by large voltage signals recorded from both the diaphragm and abdominal muscles, as shown in Figure 3. The left panels illustrate raw respiratory muscle EMG activity, whereas the traces are rectified and smoothed (time constant of 0.2 s) in the right panels. Respiratory-related activity of the muscles is evident prior to and following the retching period. These stereotyped responses served as markers for the onset and offset of an emetic episode.

Table 2 indicates the responses of the animals to CuSO_4_ administration. All animals exhibited a retching period, and in six of ten cases infusion of CuSO_4_ produced a retching response within 10 min. A linear regression analysis showed no evidence that the CuSO_4_ dose affected the time to the first retch (*p* = 0.58; *R* ^2^ = 0.04), the duration of the first retching episode (*p* = 0.48; *R* ^2^ = 0.06), or the number of emetic responses that were elicited (*p* = 0.32; *R* ^2^ = 0.12). Although numbers are small, there were also no overt differences in the emetic responses of animals of different sexes to CuSO_4_ administration.

**FIGURE 4:**
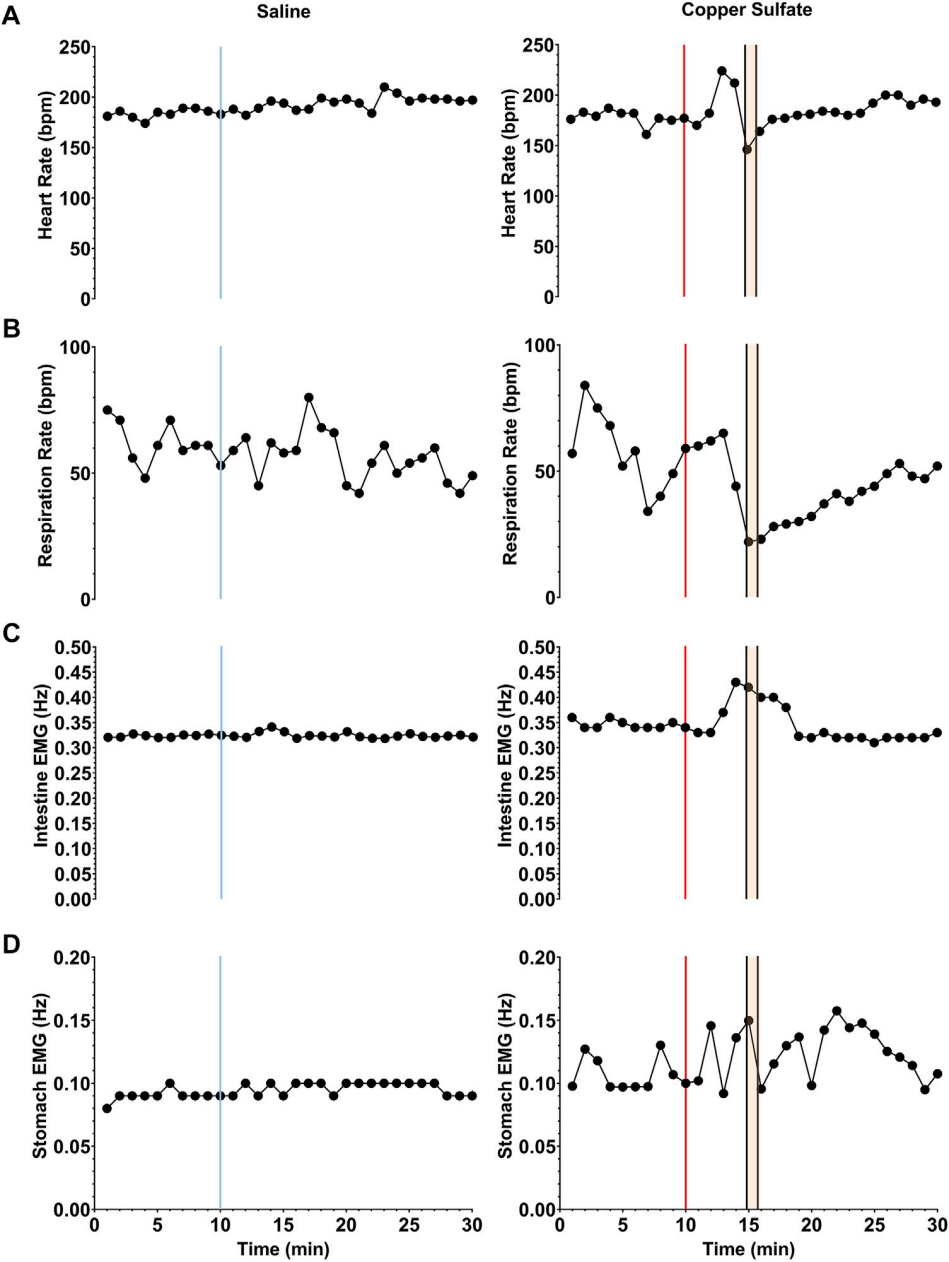
Changes in heart rate **(A)**, respiration rate **(B)**, and the frequency of slow wave activity in the duodenum **(C)** and stomach **(D)** elicited by saline (left column) and CuSO_4_ (right column) administration in one animal. Each data point indicates averaged data over a 1-s period. The vertical blue line in the left column denotes the time of saline administration, whereas the vertical red line in the right column shows when CuSO_4_ was provided. The time period when retching occurred following CuSO_4_ administration is designated by gold shading and black vertical lines.

Changes in heart rate, respiration rate, and the frequency of slow wave activity in the duodenum and stomach elicited by saline and CuSO_4_ administration in one animal (C2-21) are shown in Figure 4. Each data point indicates averaged data collected over a 1-min interval. In this animal and all others, saline administration did not elicit retching. However, within a few minutes of CuSO_4_ administration a retching period occurred, as denoted by a gold area and black vertical lines. Just prior to retching, a distinct increase in heart rate and frequency of intestinal slow-wave activity occurred. Heart rate then dropped at the onset of the retching episode, whereas the increased frequency of intestinal slow-wave activity was sustained throughout the retching period and a few subsequent minutes of recording. In addition, respiratory muscle activity decreased prior to retching and the frequency of stomach slow wave activity increased, although the baselines were less stable than for heart rate and intestinal slow wave activity, so these responses are less evident.

**FIGURE 5:**
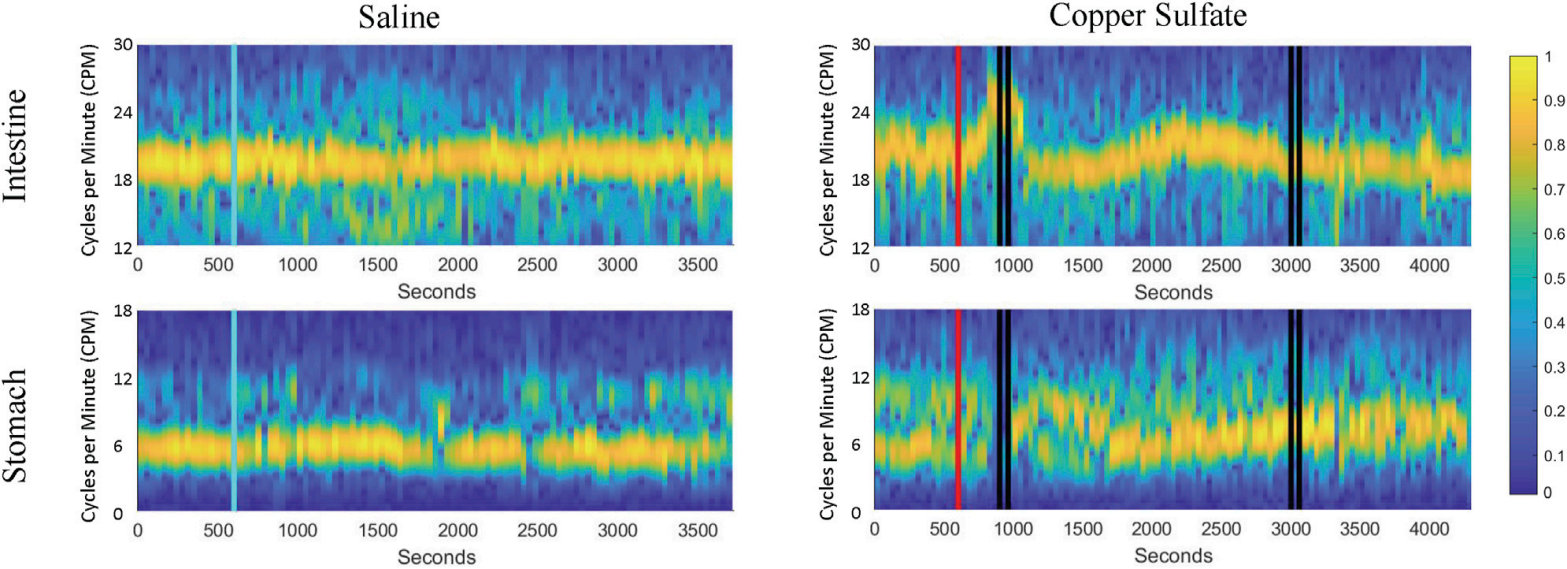
Spectrograms of duodenal and stomach slow-wave activity following administration of saline (left column) and CuSO_4_ (right column). Yellow represents the dominant frequency and blue the least dominant (scale of normalized signal power, 0–1). As in Figure 4, the blue vertical line indicates saline administration, the red vertical line designates CuSO4 administration, and the black vertical lines show when retching occurred.

To enhance visualization of changes in the frequency of stomach and duodenal muscle slow-wave activity, 1-min averages of data were also displayed as spectrograms generated using MATLAB, as illustrated in Figure 5. Data for the same animal are provided in Figures 4, 5, with a longer time period depicted in Figure 5. These spectrograms indicate that little change in slow wave activity occurred following intragastric saline administration, but a prominent change in duodenal slow wave activity commenced before and extended through the first retching period (indicted by vertical black lines). Alterations in stomach slow wave activity are also evident but are less prominent due to variability in the baseline. A second period of retching occurred approximately 30 min following the first episode (designated by a second group of black bars) but was not accompanied by appreciable changes in the frequency of slow wave activity recorded from the stomach or duodenum.

**FIGURE 6:**
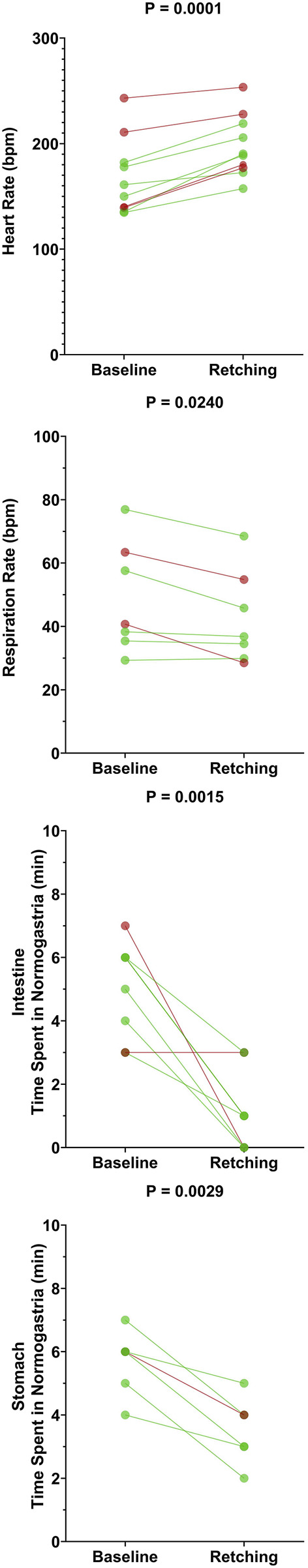
Effects of CuSO_4_ administration on physiologic parameters associated with the first retching episode. Each data point represents an average of data over 10 min during the baseline (prior to CuSO_4_ administration) or retching periods. For the six animals that retched within 10 min of providing CuSO_4_ (data indicated by green lines and symbols), the retching period was defined as the 10 min following CuSO_4_ administration. For the four animals where retching occurred at longer latency (data indicated by red lines and symbols), the retching period was defined as the 10 min prior to the final retch of the initial episode. *p* values above each panel are for comparisons between each physiologic parameter during the baseline and retching periods (two-tailed paired *t*-test).

Figure 6 compares average heart rate, respiration rate, and the span of normal intestinal and stomach slow-wave activity (normogastric activity) during the baseline period (10-min period prior to CuSO_4_ administration), and the 10-min period during which the first retching episode occurred. As in our previous study (Nanivadekar et al., 2019), normogastric activity was defined by establishing a 99% confidence interval of EMG activity during the baseline period. Average EMG activity during each minute of both the baseline and retching periods was classified as normogastric (within the confidence interval), tachygastric (above the confidence interval), or bradygastric (below the confidence interval). As indicated in Table 2, six of the animals retched within 10 min of CuSO_4_ administration. For these animals, whose data are indicated in green in Figure 6, the retching period was defined as the 10-min epoch immediately following CuSO_4_ delivery. In the four animals with delayed retching responses, whose data are indicated in red in Figure 6, the retching period was defined as the 10-min epoch preceding the final retch of the initial emetic response. Due to deterioration of recordings from some electrodes over time, data are missing from some animals. Heart rate could be determined for every animal, whereas respiration rate and intestinal EMG activity was evident in seven animals, and stomach EMG activity in six animals. Two-tailed paired t-tests were used to compare physiologic parameters in the baseline and retching periods; *p* values are provided above each panel.

CuSO_4_ administration produced a significant increase in heart rate (*p* = 0.0001) and a decrease in respiratory activity (*p* = 0.024). Heart rate increased by an average of 19 ± 11%, and respiratory activity decreased by an average of 11 ± 11%. Both intestinal and stomach EMG activity were significantly disrupted (less time spent in normogastria) by CuSO_4_: intestinal normogastric activity decreased by 73 ± 34% (*p* = 0.0015), whereas stomach normogastric activity decreased by 38 ± 16% (*p* = 0.0029). The disruption in intestinal EMG activity was particularly striking in the five animals where baseline activity was stable (normogastric ≥40% of the baseline period). In these cases, intestinal normogastric activity decreased by 86 ± 19% following CuSO_4_ administration.

A similar analysis was conducted for intragastric saline administration to the animals. Since saline did not evoke retching in any of the animals, the effects of saline on physiologic parameters were determined during the epoch defined as the retching period following CuSO_4_ administration. A two-tailed paired *t*-test showed no effects of saline administration on heart rate (*p* = 0.63), respiration rate (*p* = 0.83), or disruption of normogastric intestinal (*p* = 0.61) or stomach (*p* = 0.60) activity.

## 4 Discussion

This study incorporated extensive monitoring of physiologic parameters in an alert animal model during the administration of an agent that produces rapid emetic events, allowing the analysis of prodromal physiologic changes that precede vomiting. The frequency of baseline GI myoelectric activity noted in this study is similar to that documented in prior experiments (Connor, 1979; Roche et al., 1982; Lang, 1999). CuSO_4_ infusion elicited a significant increase in heart rate, decrease in respiratory rate, and a disruption of gastric and intestinal EMG activity several minutes prior to emesis. The most striking and consistent prodromal physiologic changes were an increase in heart rate and a reduction of time that duodenal activity was normogastric. In contrast to the prevailing focus of work showing changes in the gastric EMG signals after emetic stimulation (Koch et al., 1990; Uijtdehaage et al., 1992; Kiernan et al., 1997; Parkman et al., 2003), our work shows that EMG activity changes were most consistent in the duodenum. It is postulated that intestinal EMG activity was related to a retrograde movement of chyme from the intestine to the stomach, which could be associated with the prodromal retrograde contractions reported after emetic treatments (Lang, 1999; Ueno and Chen, 2004; Koch, 2014; Heyer et al., 2018).

A low dose of 83 mg CuSO_4_ was used in this study, as this has been a standard dose in prior experiments (Sugiyama et al., 2011; Suzuki et al., 2012), and reliably produced emesis in this study. Although threshold doses of CuSO_4_ for producing nausea and retching have not been established in humans, a literature review suggested that a dose as low as 15 mg could produce gastric distress (Franchitto et al., 2008). Higher doses were also used to ascertain whether they elicited different responses than lower doses. Increasing the dose of CuSO_4_ did not appear to alter the physiologic effects of the treatment or the length of time between the administration of the agent and the first retching event. For example, 83 mg doses produced retching in 6–14 min, whereas 332 mg doses produced retching in 4–33 min. These findings suggest considerable individual variability in gastric responsiveness to CuSO_4_.

Administration of 20 mL saline in a preceding recording session (on a different day) did not induce similar physiologic changes as CuSO_4_, indicating that they were not induced by a distension of the stomach with fluid. Systematic changes in heart rate and disruption of GI myoelectric activity only occurred within a short period preceding and during emesis (see Figure 4). These observations suggest that the changes in heart rate and GI myoelectric activity observed in the study were related to nausea, and not non-specific actions of CuSO_4_ such as irritation of the stomach lining.

Several caveats and limitations of the current study should be noted. First, in some cases we were not able to administer CuSO_4_ through the implanted gastric tube, and instead gavaged CuSO_4_ solution. Although the physiologic effects in these animals were similar to those in animals that received the agent intragastrically (see Table 2), it is possible that the distribution of CuSO_4_ in the stomach differed between the routes of administration. Moreover, in general there was variability of stomach geometry and placement of the tip of the delivery tube in the gastric compartment; therefore, it is difficult to determine the specific chemical placement and timing of movement of the emetic agent along the GI tract. In addition, we also did not attempt to replicate the CuSO_4_ tests within each animal because CuSO_4_ is known to damage the GI mucosal surface (Chuttani et al., 1965; James et al., 1999; Nastoulis et al., 2017).

This issue of testing repeatability is likely important to translate these findings for potential clinical application. Although acute emesis (and nausea) can be controlled by administration of currently available antiemetics, such as 5-HT3 and NK-1 receptor antagonists, the chronic nausea and vomiting that occurs in GI disease is more difficult to control (Hasler et al., 2017; Lacy et al., 2018). Potentially, these chronic clinical conditions could be modeled with a repeated testing paradigm using pre-clinical models. Importantly, this work also needs to be extended to include other emetic stimuli, acting on non-vagal pathways, including motion and those that activate area postrema. Nonetheless, the current findings provide a blueprint for these future studies by highlighting the importance of monitoring intestinal EMG activity. It is also possible that a machine learning paradigm that incorporates changes in heart rate and disruption of intestinal myoelectric activity would provide a particularly effective method of predicting the onset of emesis.

## Data Availability

The raw data supporting the conclusions of this article will be made available by the authors, without undue reservation.
